# Very low frequency waves as selective probe for *Cysticercus tenuicollis*, *Hydatid cyst* and *Coenurus cerebralis* bio-analysis using single cell-signal recording

**DOI:** 10.1038/s41598-022-20456-5

**Published:** 2022-11-22

**Authors:** Hamed Foroutan, Mohammad Moazeni, Mohammad Mahdi Doroodmand, Amir Mootabi-Alavi

**Affiliations:** 1 Department of Pathobiology, School of Veterinary Medicine, Shiraz University, Shiraz, 71345-1731 Iran; 2 Department of Chemistry, School of Sciences, Shiraz University, Shiraz, Iran

**Keywords:** Electrophysiology, Biochemistry, Biological techniques, Biophysics, Diseases

## Abstract

Comparative electric behavior of *Cysticercus tenuicollis*, *Hydatid* cyst and *Coenurus cerebralis* at the Very Low Frequency (VLF) region has been studied in detail. This investigation could be significant, because of the economic and public health importance of these parasitic infections in domestic animals. In this report, a single cell signal recording technique has been adopted for comparison using a stainless steel (type: 316, diameter: ~ 300 µm, height: 2.00 cm) two identical electrode system, implanted on the surface of the tested cysts with inter electrode distance of 0.50 cm at a ~ 6.0 giga ohm (GΩ) sealed condition (based on the situation of the implanted electrode system). This process was achieved based on applying electrical interaction between the cysts and the VLF electrical signal. Relative to the measured time domain signal (Current–time diagram), the frequency domain (Current-frequency diagram) was estimated via applying a “Discrete Fast Fourier Transform” (DFFT) algorithm at a fixed time interval (5.0 min). Factors, having important influence on the sensitivity of the detection system including the type (waveform) of different alternating-current (AC) triggering stimulus signals (such as direct current, square wave, triangular, sin (t), etc.), the amplitude, as well as the frequency were optimized automatically through a written “*Visual Basic 6*” program by one-factor-at-a-time method. Direct applying this AC triggering VLF voltage to the cysts resulted in tracing an AC electrical current vs. time that considered as the time domain wave. However, this electrical current was decayed rapidly versus time during maximum 30.0 s time scale. Applying the DFFT algorithm to the measured time domain, resulted in accessing to the frequency domain at the selected frequency range between 2 and 5 kHz that was considered as the selected frequency for the selective differentiation of *C. tenuicollis, Hydatid cyst and C. cerebralis*. The related probable mechanism of this process may be attributed to the correlation between the triggering potential and the cyst’s electrical surface charge (Zeta potential) as the current source under similar conditions. The results of this study may help to introduce a new detection system for in vivo recognition of the cysts in future.

## Introduction

*Taenia hydatigena* (*T. hydatigena*) is a widespread parasite that lives in the small intestine of dogs, cats, foxes and wolves. *Cysticercus tenuicollis* (*C. tenuicollis*) is the larval stage of the *T. hydatigena*. *C. tenuicollis* may be observed in a wide range of mammalian hosts such as sheep, goat, camel, cattle, dear, pig, boar, rat and monkey, however, it mainly occurs in sheep and goats. The cyst has a translucent wall and contains a scolex, neck and a sac that is filled with fluid^1–6^. Mature cysts of *C. tenuicollis* are mostly observed while they are attached to the mesentery, omentum, peritoneum, liver, urinary bladder, pericardium, chorio-allantoic membrane, pleura, lungs, diaphragm, pelvic cavity, brain, uterine tubes, ovaries, uterus, vagina and cervix^4,5,7–15^. Generally, the infections are chronic in nature and asymptomatic, hence, could not be identified before slaughter^16^. The parasite may cause reduction in production and economic losses due to condemnation of liver and other infected organs^5,6,17–22^.

*Echinococcus granulosus* (*E. granulosus*) is a small tapeworm that lives in the small intestine of dogs and other canids^23^. Hydatid cyst is the larval stage of *E. granulosus*. It is a space occupying lesion, that develops in various organs, particularly in the liver and lungs of animals as well as human beings^24,25^. One hydatid cyst may grow as much as a cyst containing several liters of hydatid fluid^26,27^. Clinical signs depend on the number, size, and location of the cysts^28^. Even though the disease may be asymptomatic, it can be fatal in human beings^29^. The mortality rate of cystic echinococcosis (CE) could reach to 4% but it may rise significantly in poor medical conditions^30^. Diagnosis of hydatid cyst is based on clinical signs, imaging (Radigraphy, ultrasonography, CT scan, MRI), and serology^31^. Surgery is considered as the main treatment option for the disease, however, chemotherapy may be the only treatment option during pregnancy, for multiple, very small or inaccessible cysts and in old people as well as in those who do not accept surgery ^24,25,32^.

*Taenia multiceps* (*T. multiceps*) is a taeniid tape worm living in the small intestine of dogs and other carnivores. The larval stage of *T. multiceps* is commonly name *Coenurus cerebralis* (*C. cerebralis*). It is a large, delicate, thin, translucent and fluid containing cyst, measuring about 5–6 cm in diameter. In addition, a large number of scolices (400–500) appearing as white clusters are attached to the internal layer of the cyst wall. The cysts may be found in brain, spinal cord, and to a lesser extent in intramuscular and subcutaneous tissues^33–35^. Coenurosis (infection with *C. cerebralis*) is a fatal parasitic disease of the central nervous system (brain and spinal cord) of domestic and wild ruminants, pigs, horses and humans^36^. The disease has a worldwide distribution^35^. The clinical signs of coenurosis, depend on the number, location and developmental stage of the cysts as well as the host immune response^37^. So, they are variable and nonspecific. Therefore, the clinical diagnosis is complex. Hence, more specific and reliable diagnostic tools are required. However, in humans, diagnosis is based on the identification of cysts in the brain by magnetic resonance imaging (MRI) and computerized tomography (CT) scan^38^. When clinical signs of coenurosis were appeared, the prognosis of the disease is extremely poor and fatality rate may reach to 100%. Hence, there is little or no success in chemotherapy. Therefore, surgical removal of the cyst remains as the only option. However, surgical operation is limited to economically or genetically valuable animals and is not advisable in field conditions^33^.

The VLF wave, similar to other regions of electromagnetic waves, possesses both electrical and magnetic components. But, comparing to other electromagnetic frequencies, the VLF region seems to be more effective for the bio-sensing/analysis purposes. Because of its small frequency, and consequently its low energy, has no side effect(s) for both animals and human beings.

Even though, the above cysts are easy to detect after necropsy of the dead animals, their detection in live animals requires ultrasound or other advanced imaging techniques such as CT scan or MRI that are expensive, scarcely available and not applicable in ordinary clinical practice.

Immunodiagnostic as well as molecular tests also have several limitations. Immunological tests like Enzyme-Linked Immunosorbent Assay (ELISA*)* methods are time-consuming, not available in all laboratories, suffer from cross-reactions, are highly dependent on the purity of antigens used, are limited in sensitivity and specificity, have laborious procedures and need skilled operators and spectroscopic instrumentation. Molecular assays, such as Polymerase chain reaction (PCR) also has limitations including high contamination risk, cost, lack of quantitative results and non-availability in many centers. Therefore, in addition to the present conventional diagnostic tools, there is a need to introduce novel and reliable methods for diagnosis of the mentioned cysts in animals and especially in humans. This study was performed to compare the electric behavior of *C. tenuicollis*, Hydatid cyst and *C. cerebralis* at the VLF region. The results of this study may help to introduce a new detection system for direct recognition of the cysts in future.

## Experimental

### Collection of the cysts

*Cysticercus tenuicollis*, hydatid cyst and *C. cerebralis* were obtained from the naturally infected sheep slaughtered at Shiraz slaughterhouses. *C. tenuicollis* was collected from mesentery, peritoneum and liver, hydatid cysts were collected from the liver and *C. cerebralis* was collected from the infected muscles of slaughtered sheep. After collection, the cysts were transferred to the parasitology laboratory under sterile and cold conditions. The cysts were then freed cautiously from the surrounding tissues and were washed with distilled water twice at room temperature. Finally, the electrode system was directly implanted onto the cysts wall for the further comparison and analysis.

### Instruments

**Figure 1:**
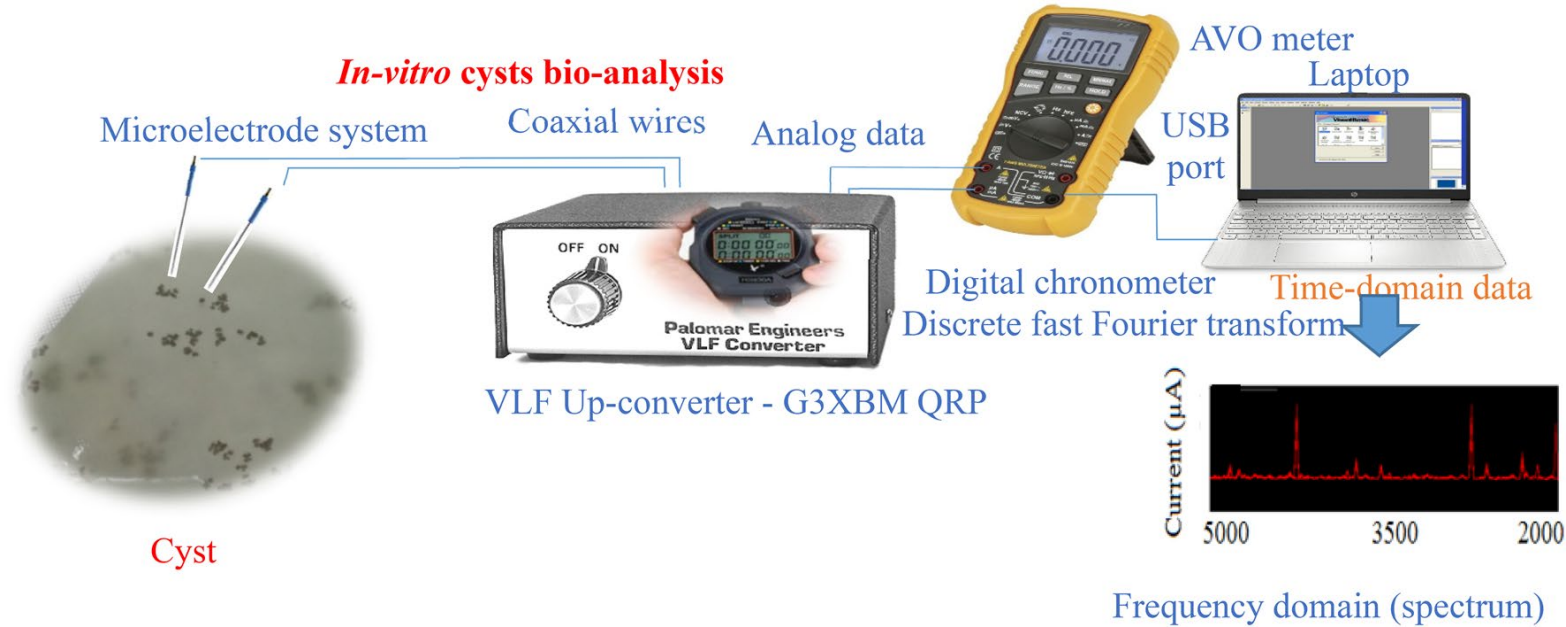
Schematic of the designed and introduced instrumentation system for cyst bio-analysis process.

Schematic of the designed and introduced instrumentation system for the cyst bio-analysis purpose has been shown in Fig. 1. As clearly exhibited, the calibrated instrumentation systems were selected for the efficient detection and recognition of the cysts (Table 1). As shown, some different calibrated instruments, noise shielded coaxial wires and various types of electronic elements such as resistors and capacitors with military series were adopted to operate the current cyst bio-analysis process.

**Table 1:** List of adopted instrumentation system used for the comparative investigation between the cysts behavior at the VLF region.

No	Instrument	Deception/company
1	Digital voltmeter	Fluke 287, USB Port, equipped with Fluke IR189USB USB Cable Adapter, Digital Megohmmeter Insulation Resistance Tester, USA
2	Cubic faraday’s cage	Dimension: 50.0 × 50.0 × 60.0 cm, stainless steel, Type: 308
3	Digital micrometer	Mitutoyo 395–353—MIC, Series 395, US
4	Electrical resistance	R = 1.000 Ω, Military Series (*RLR*) metal film resistors, MIL-PRF-26, USA
5	Resistor	R = 1.000 kΩ, Military Series (RLR) metal film resistors, MIL-PRF-26, USA
6	Coaxial wires	RG6, CIMPLE CO—30' Feet, USA
7	BNC connector	China

It was noticeable that, selection of electronic elements with the military series was based on dealing with electronic components with maximum reliability and minimum tolerance. In this system prior the analysis, all the modules of the instrumentation system were therefore calibrated and standardized using a serial resistance–capacitance (Serial R–C elements, military Series, 1.00 kΩ, ad 150.0 µF, Analog device) as dummy cell. For this purpose, the probes (wires) were directly connected to the R–C electronic circuit instead of the cysts body through the implanted electrode). At this condition, not only the researcher would access to full confidences about the accuracy of the electronic systems, but also, was able to calibrate all parts of the electronic modules such as the gain (sensitivity) of the amplifier and the average of the signal-to noise (S/N) ratio. The signal acquisition process was also adopted using the fluke tester through the USB port. Due to the effective role of the adopted and optimized VLF electrical signal on the cyst bio-analysis process, the experimental section about this waveform was evaluated with detail (Table 2). As clearly exhibited, the frequency range as well as the optimum values of the VLF wave were clearly reported. These factors were automatically provided via programming the assembled VLF function generator.

**Table 2:** Detail of the assembled VLF function generator used for the comparative investigation between the cysts behavior at the VLF region.

No	Instrument	Deception/model/company
1	VLF signal generator	VLF Up-converter—G3XBM QRP
2	Peak-to peak voltage (V_p–p_)	+ 100.0 mV (vs. the pseudo reference probe)
3	Optimum excitation potential signal (waveform)	Square wave waveform
4	Triggered (applied) potential	1.5 V, DC (vs. the pseudo reference probe)
5	Applied electrical frequency (VLF)	2–5 kHz
6	Electronic potentiostat	Analog device
7	Duty cycle	50%

Prior the analysis, all the modules of the instrumentation system were calibrated and standardized using a resistance–capacitance (R–C, military Series, 1.00 kΩ, ad 150.0 µF, electrolyte, Analog device) dummy cell.

It should be noted that, in all parts of the experiment the electrical potentials have been applied versus the total applied potential. Electronically, it is impossible to apply absolute potential to any electronic/device systems. This is correlated to the definition of the absolute electrical potential/voltage that points to the energy needed for transferring of a unit electrical charge (1 C) from one region to the infinite point. Consequently, for simplicity of the experimental systems, the electrical potentials are applied based on the potential/voltage gradient (difference) between a point and a fixed (reference) condition such as total applied potential. At this condition, it is exponentially possible to evaluate and compare the potential energy vs. the pseudo reference probe.

### Single cell signal recording methodology: Electrode implant

To estimate the electrical behavior of each cyst, two identical stainless steel (Type: 316) electrode system was included as working and pseudo reference probes. For this purpose, the needle of commercial medical micro-syringe (Insulin type) was suitable. These needles were directly connected to the AVO-meter probes simply using an electronic BNC micro-connector. Then, the conductive surfaces of both needle and probe were isolated using a tubing thermal shrink. At this condition, only around 2.00 cm height from the tip was isolated, which provided the electrical signals during the bio-analysis process.

Each electrode was then gently (due to the softness of the tested tissue) implanted onto the surface of each cyst by hand. The inter-electrode distance was fixed at 0.50 ± 0.01 cm. In addition, to have full confidence about the electrically dis-connectivity of the adopted VLF function generator at the switching off step, high impedance mode was selected. This mode was provided via providing giga sealed condition on the bio-analysis system. This was achieved during applying electrical resistivity ~ 6.0 GΩ depending on the situation of the implanted electrode system.

A constant direct/alternating current (*DC/AC*) potential (voltammetry, or single cell signal recording process) was applied to the working electrode versus the pseudo reference micro-electrode. Then, the potentiostat electrical parameters were measured on the surface of the working electrode (versus the pseudo reference electrode). The reason behind this bio-analysis using the “*Single Cell Signal Record”* was related to the unclear and randomized electrical signal acquisition from the cysts.

### Procedure

After introducing the two-electrode system onto each tested cyst, the VLF signal generator was set at V_P–P_ of +100.00 ± 0.01 mV (vs. the pseudo reference probe) as the waveform of square wave during frequency scanning (sweeping) between 2 and 5 kHz. This frequency range was adopted to access the cyst’s spectrum. Obviously, selection of a frequency range for the cyst recognition purpose was based on the direct observation of peak/shoulder at a certain frequency region during introduction between the cyst body and the electrical waveform. The VLF waveform was modulated onto a fixed triggered DC potential source as large as +1.50 ± 0.01 V (vs. the pseudo reference probe) as both the cyst triggering and frequency adjusting potential. Then, the signal generator was turned on to electrically apply the electrical stimulus through the electronic potentiostat circuit along 5.0 min time interval. Afterward, the signal generator was turned (switched) off and the *AC* current data were sampled versus time along the 30.0 s period of time until decaying to the zero current using the digital AVO meter. The data were then transferred to a PC through the USB port and 999.2 software (Version: 6.2) and stored inside an excel file.

### Fourier transform algorithm: detection of the frequency domain

The access to the electrical spectrum, the time domain diagram was converted to the frequency one using the Discrete Fast Fourier Transform process through a written software^39^. This process was achieved inside a PC via separating the real and imaginary phases, which processed 90 phase gradients. The sin (t) and cos (t) phases, interpreted these two phases, can therefore be considered as selective probes for further bio-analysis of the selected cysts.

### Zeta potential: surface excess charge of the tested cyst

The *Zeta* potential of the cyst containing water fluid (5.0 mL) was evaluated using a *Zeta* potential analyzer (Zeta-Meter, Inc. PO Box 3008, Staunton VA 24402 USA). For this purpose, the electrode system was again introduced onto the fluid blindly analog with estimation of the Zeta potential (vs. the pseudo reference probe) according to a published procedure^40^. This method was therefore considered as acceptable probe for evaluation of the reliability of this bio-analysis system.

### Statistical tests

All the results were the average of at least four sequential analyses. The uncertainty of each datum was based on the ± standard deviation (SD) with three degrees of freedom and 95% confidence level. The electrical parameters were optimized by the one-factor at a time method automatically through a program written in Visual Basic 6 (*VB*_*6*_) for reaching to maximum current gradient sensitivity. The reliability of the results was evaluated based on the t-test method with two degrees of freedom.

### Electrode system introduction: Giga ohm sealed condition

**Figure 2:**
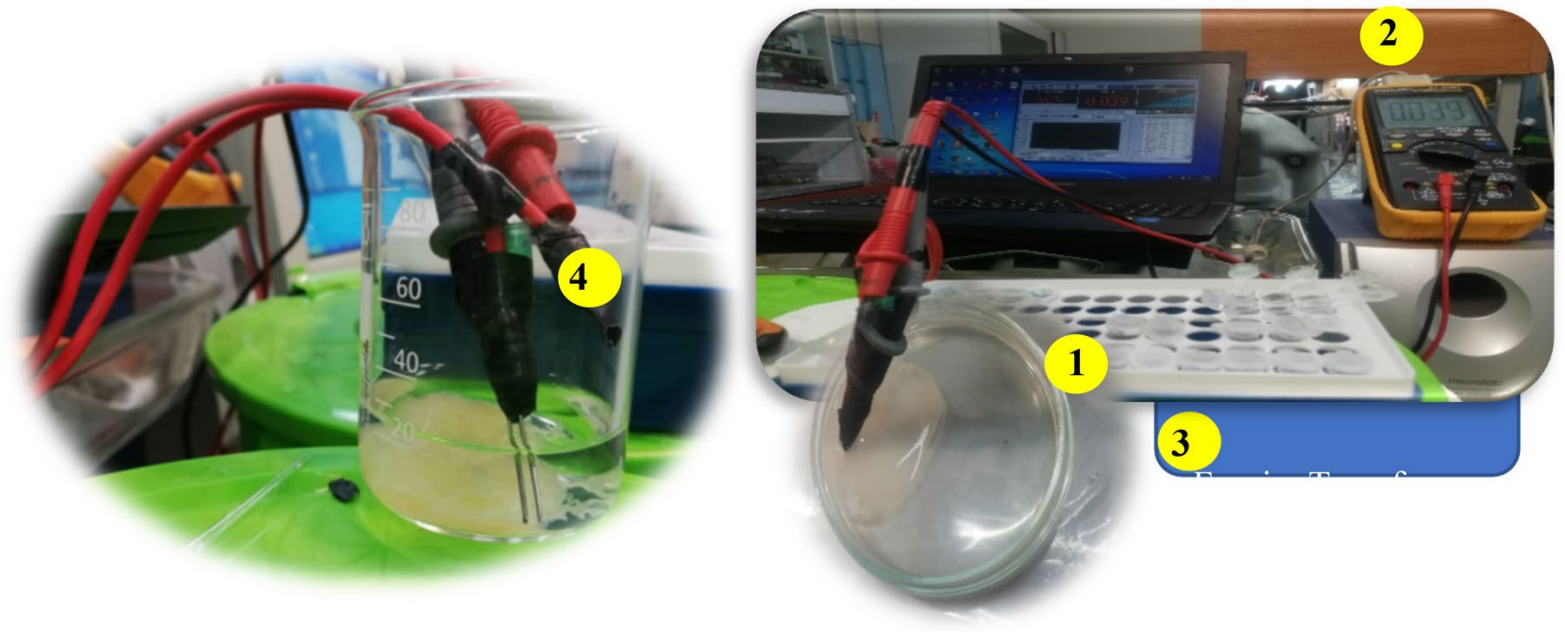
Adopted electrode system, implanted onto the cysts. (1) Petri dish containing cyst, (2) AVO meter, (3) DFFT algorithm and (4) Configuration of the adopted electrode system.

To apply the electrical current, a two identical electrode system including implanted working and pseudo reference electrodes with inter electrode distance of 0.50 ± 0.01 cm (Fig. 2) was adopted to the system. Configuration of the adopted electrode system has also been shown in Fig. 2 (*Part 4*).

In this method, although the electrode system has been directly implanted onto the cysts, nonpolar behavior of the membrane of the tested cysts (almost due to the phospholipid layers of the cysts^41^, caused to deals with high internal resistance above 10 mΩ. This condition provided huge ohmic potential (*IR* drop, high impedance mode) for the cysts. Consequently, this situation resulted in significant polarizability of the tested cysts. As the results, the cyst showed rapid electrical response to the external electrical field at a fixed time interval (5.0 min).

### Data acquisition system

After applying (triggering) the electrical current based waveform through the two-electrode system with the fixed inter-electrode distance (0.50 ± 0.01 cm), naturally at a giga ohm sealed conditions (~ 6.0 GΩ), at a fixed time interval (5.0 min), the cysts were electrically excited (stimulated). The exciting process may be attributed to different parameters such as surface electrical charge/discharge, pseudo capacitive (dielectric) behavior of the cysts, surface polarization of the cysts, etc. This excitation (induction) behavior was survived even after turning off the electrical stimulating currents that was decade freely during at least 2.0 min time interval. This current–time response was named as Free Induced Decay (FID), trace and/or time domain.

### Parameters affecting the bio-analysis system

**Figure 3:**
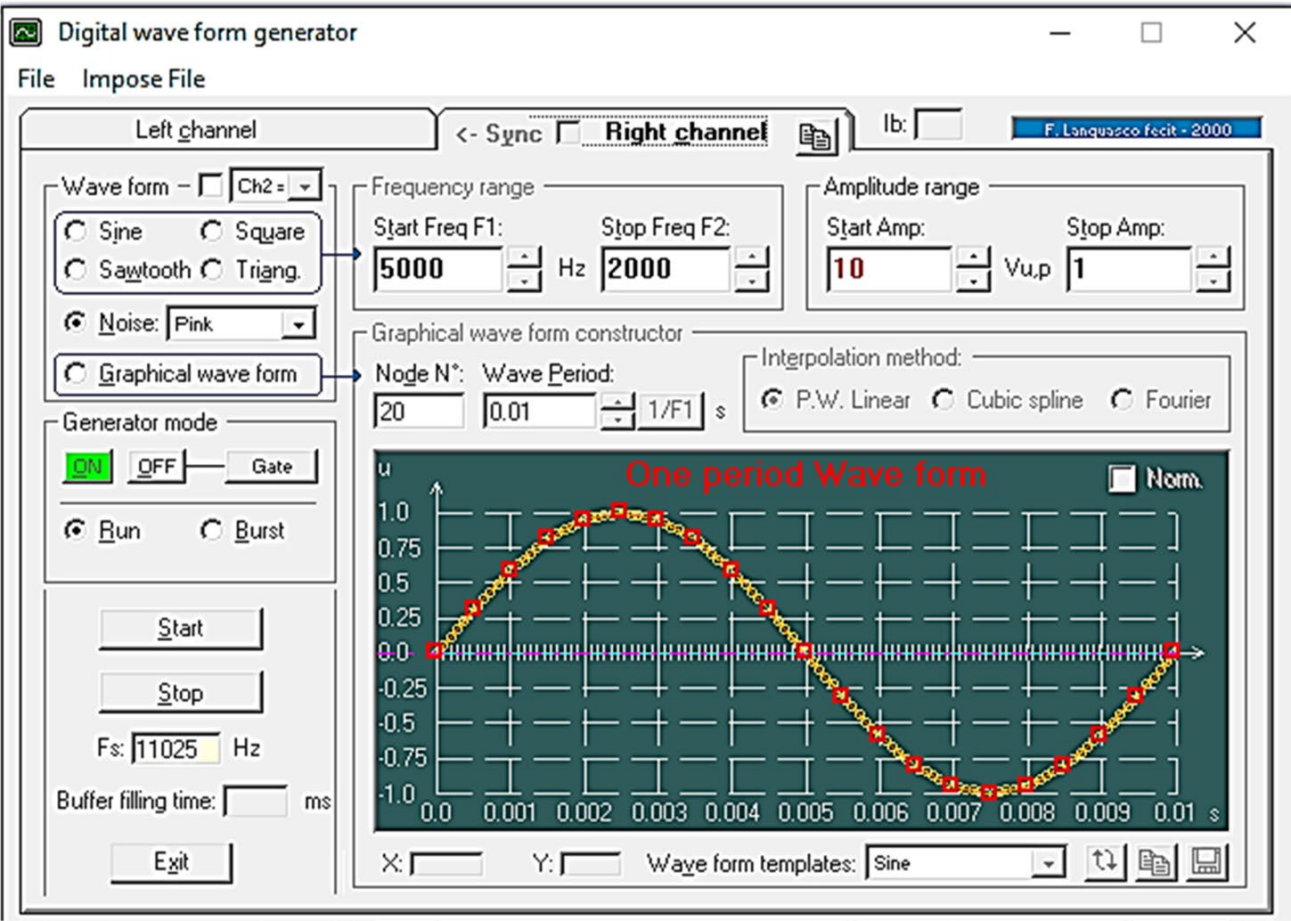
Schematic of the lab-written Visual Basic 6 (*VB*_*6*_) software to generate electrical current based waves.

The selected current-based excitation signal (waveform), the square wave as well as the related parameters such as the frequency as well as the initial clamp current parameters having important effect on the sensitivity of the detection system has been graphically shown in Fig. 3.

As shown in Fig. 3, the written software was capable to generate different current-based waveforms (expiation signals) via direct triggering the electronic system as a function generator with electrical frequency almost at the VLF region. The amplitude (intensity) of the wave could also be automatically controlled through the software. It should be noted that, the designed electrical function generator was consisted of five different channels that was able to operate simultaneously and independently different waveforms with distinct electrical characteristics. The generated waveform can be applied to the cysts structure through a Galvanostat and a USB port.

### Ethics committee

This studied was admitted and approved by the ethics committee of the Shiraz University Consul.

## Results and discussion

Based on the electro-stimulating evidences^42^, the electromagnetic waves, due to possessing both electrical and magnetic components, are selected as good candidate for the detection purposes^43^. However, morphological characteristics (speciation) of the cyst strongly depends on differentiation between the electrical frequencies. This feature is considered as the most important factor for approaching to this aim. To the best of knowledge, no probe has been reported in the literature review for accessing to these differentiated frequencies. In this research, for the first time the contribution between chemistry and parasitology has been resulted in accessing to a novel methodology for the cysts detection process. Detail of this bio-detection system has been discussed in the following sections.

### Time domain

**Figure 4:**
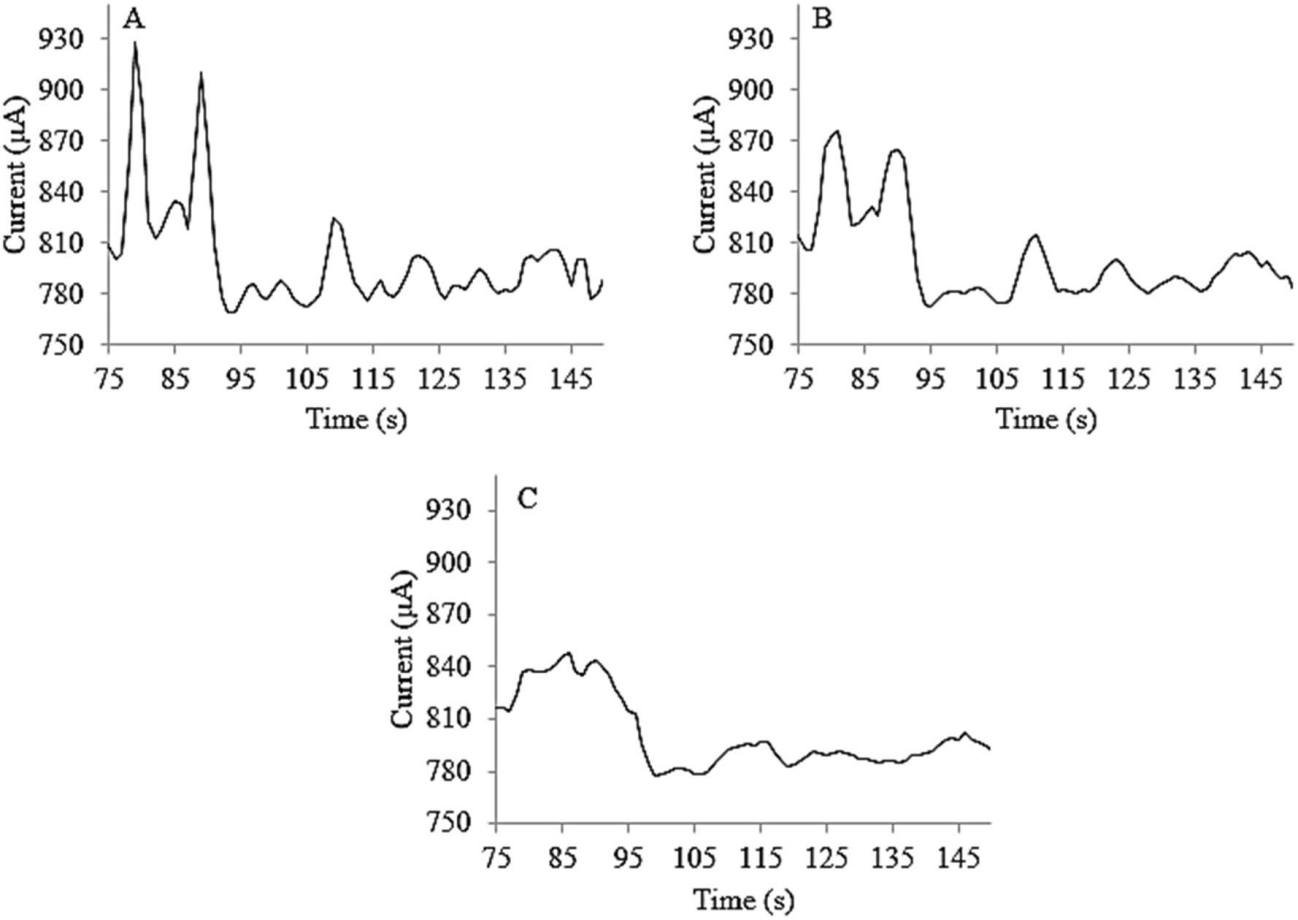
Time domains (free induced decay, current–time diagrams) of (**A**) *C. tenuiculis*, (**B**) *ydatid cyst* and (**C**) *Coenurus cerebralis* after applying the Sin(t) waveform. Triggered electrical potential: + 1.50 ± 0.01 V (vs. the pseudo reference probe). Time interval: 5.0 min. Inter-electrode distance: 0.50 cm, frequency step: 2 Hz.

The traces (time domains) of the results have been shown in Fig. 4. It also should be noted that, to promote the sensitivity of the time domain responses as well as to eliminate any type(s) of negative/positive bias errors during estimation of the cyst interacted frequencies (especially when promoting the signal-to- noise ratio by signal averaging process during the multiple (at least three scanning processes, n = 3, frequency step: 2 Hz), the VLF waveforms should be modulated onto a fixed triggered DC electrical pulse. This pulse was therefore accepted as both the cyst triggering and frequency adjusting potential. Based on the results (Fig. 4), maximum sensitivity was observed when applying electrical pulse as large as + 1.50 ± 0.01 V (vs. the pseudo reference probe).

As shown in Fig. 4, the current decays have been measured after the end of the stimulation to discriminate the cysts, at which the cysts response freely. More reproducible (higher precision) responses were detected at this condition vs. other circumstances such as signal measurement during the stimulation. This was probably correlated to the random and irregular cyst’s perturbations during signal measuring at the time interval of the applied electrical stimulation.

### Discrete fast fourier transformed detection system

**Figure 5:**
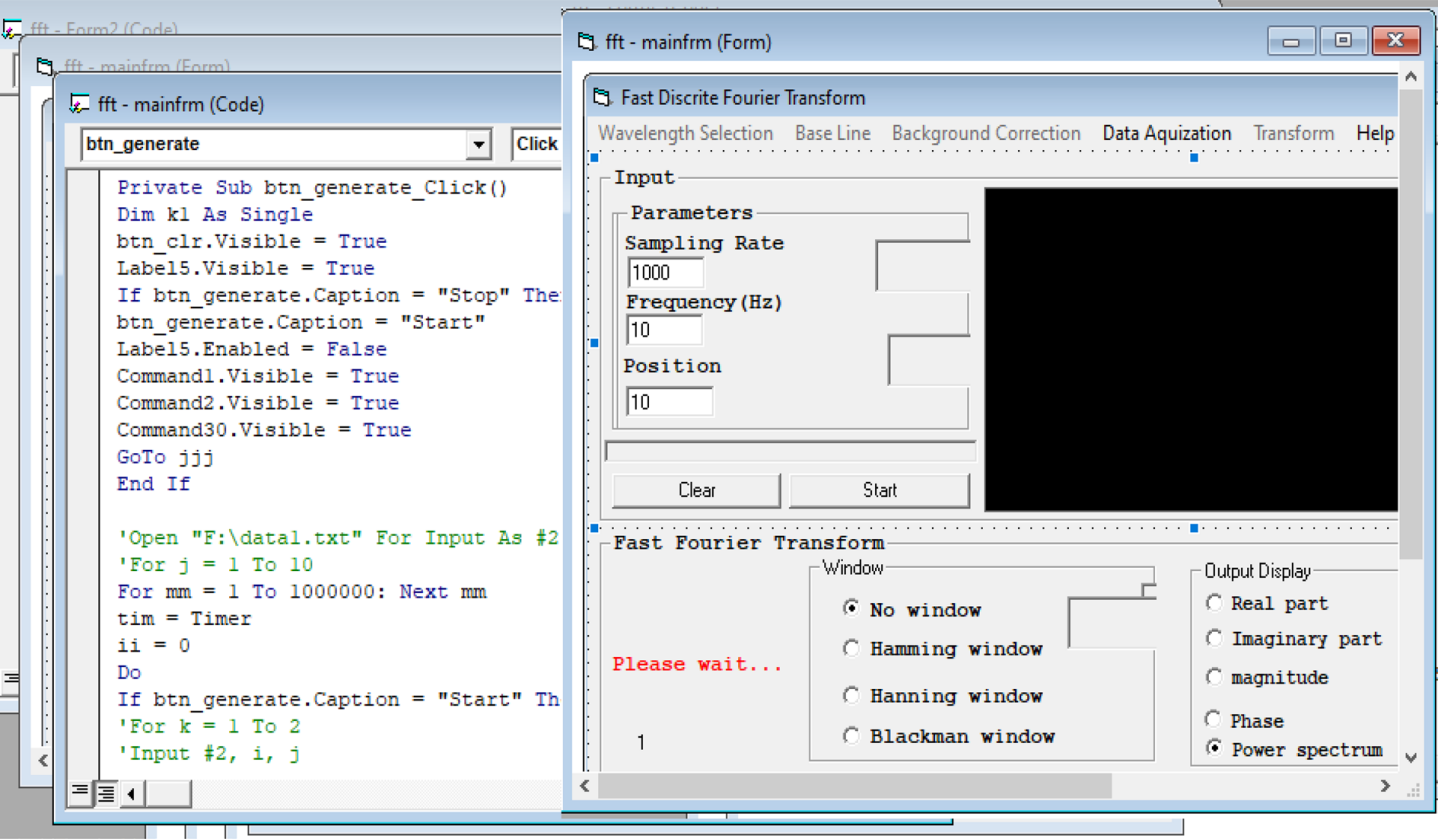
Written Visual Basic 6 (*VB*_*6*_) software to apply the DFFT algorithm to the time domain signal.

This time domain (trace) data was then transferred to a PC for applying with the *DFFT* algorithm through a written program in VB_6_ software (Fig. 5).

### Optimization process

Parameters having important effect(s) on the sensitivity of the detection system include, the type of the AC voltage triggering waveform, the amplitude, electrical frequency, and time duration of the applied potential. However, at the initial stage, it was suggested to optimize the mentioned parameters by one-factor-at-a time method, but special cases were detected for the mentioned parameters that are evaluated in detail in the following sections:

#### Selection of waveform (potential excitation signal)

Comparing to different waveforms such as DC, square wave, triangular and sin (t) waveforms, only square wave with 50% duty cycle was suitable for having effective interaction with the tested cysts. In another word, no major (significant) changes were observed in each of time- or frequency domain, during applying different fixed current (Galvanostat) or constant potential (Potentiostat) at a fixed time interval (5.0 min). About other tested waveforms, the detected results were so irregular and random that, practically, it was impossible to have any judgment about reliability of the results. The adopted waveform was therefore selected as a harmony waveform for efficient interaction with the tested cysts as the selected probe.

The harmony of the selected square wave waveform was probably attributed to some different factors especially (i) effective role of this wave on the sensitivity of this detection system (possibly due to the rapid and sequential stimulation of the cysts during clocking the wave at two high and low potential levels), (ii) electronically, small distortion of the square wave (vs. other waveforms), (iii) ease of modulation of the this VLF wave with a DC voltage , and (iv) facility applying *DFFT* algorithm on the demodulated VLF waveform during estimation of the frequency domain (spectrum). Consequently, this waveform was selected as the excitation signal for this detection purpose. However, it should be noted that, different on/off triggering of the square wave waveform or even clamping (cutting-off) it with different frequencies did not cause any significant influence(s) on the detection response. Consequently, this waveform was selected as the excitation signal for this detection purpose. However, it should be noted that, different on/off triggering of the sin (t) waveform or even clamping (cutting-off) it with different frequencies did not cause any significant influence(s) on the detection response.

#### Current/voltage source of the selected excitation signal

Relative to the voltage mode, large resistivity of the selected cysts that naturally resulted in dealing with the conditioned giga ohm sealed condition, using potentiostat system (during applying a fixed potential-based waveform) majorly reduced the repeatability, reproducibility as well as the sensitivity of the detection system. In addition, higher values for the amplitude of the applied electrical potential caused to regard with some perturbations during the signal processing on the surface of the tested cysts. However, some probably events such as the danger of the deactivation, denature and/or even change in the morphology of the structure (possibility due to its death) majorly limited the use of the Galvanostat mode. Consequently, it was decided to use a fixed potential at the Potentiostat mode for the excitation of the cysts at the fixed time interval. The results revealed at least a 15-fold excess in the output sensitivity (potential gradient) when applying a potentiostat mode, compared to other Galvanostat ones. Consequently, in this experiment at fixed potential-based (square) waveform has been applied to the cyst body using the implanted electrodes through the potentiostat electronic circuit; and as the results, the electrical current as time domain (FID) was measured for applying the DFFT algorithm.

#### Frequency range

Based on this hypothesis that electrical and magnetic component of the VLF waveform often caused to have acceptable electromagnetic wave diffusion along different environments^44^; high absorption capacity of the water medium as main molecule in the cyst’s structure limited the applied frequency domain. Besides this, the higher was the frequency domain, the more intensive electrical wave can be generated. This phenomenon majorly promoted the detection sensitivity and enhanced the signal-to-noise ratio (*S/N*) during reaching to more reproducibility detection process. All the information caused to select a wide frequency range between 2 and 5 kHz for the analysis process. The results also revealed that, no significant interaction(s) and as the results no sensitive electrical peak(s) were observed when applying different frequencies lower than the selected range.

#### Waveform amplitude

To optimize the potential amplitude, from one way, the higher was the potential amplitude, the more sensitivity detection system would reach; whereas from the other hand, probable side effect(s) during applying very high electrical voltages to the detection system would damage (shock) the cyst structure. Consequently, in this research, it was decided to apply electrical potential via applied a potentiostat waveform with maximum + 1.50 (± 0.01) V potential level (vs. the pseudo reference probe) using a function generator with maximum 100 W electrical power onto the wall of the tested cysts using a two-electrode system.

#### Time duration of the applied potential based excitation signal

In this study, the results revealed that, to have enough sensitivity, at least three sequential FIDs were necessity. To provide stable conditions for the tested cysts, the junction potential between the electrode surface and the cyst wall seemed to be important. To reduce this interfacial potential, the system was conditioned via staying at least 10 s before any data acquisition. Then, after accessing averaged time domain (three sequential FIDs), it was transferred into a PC for applying the mathematical algorithm to receive the frequency domain (spectrum). To promote the sensitivity as well as the *S/N* ratio, it was necessary to average the data during multiple scanning (sweeping) processes (n = 3). This process resulted in access to more sensitive signal with amplifier coefficient of *n*^1/2^, where “*n*” is the number of FIDs.

In another word, under the selected reproducible conditions, multiple scanning (sweeping) the applied VLF waveform at different frequency regions, and as the results, averaging the frequency domains again enhanced the *S/N* ratio with the n^1/2^ order. Based on the results, about three replicate analyses were needed to reach high enough sensitive and steady-state conditions. Consequently, in this study, most of the analyses were repeated at least three times under similar conditions. Obviously, at this condition, the importance of the selected cyst triggering and frequency adjusting potential on the reproducibility of the averaging process was further evidenced. All these results exhibited the importance of the selected algorithm on the noise analysis process and subsequently for dealing with highly sensitive, significant and reproducible results during the cyst bio-analysis process.

#### Discrete fast fourier transform algorithm

**Figure 6:**
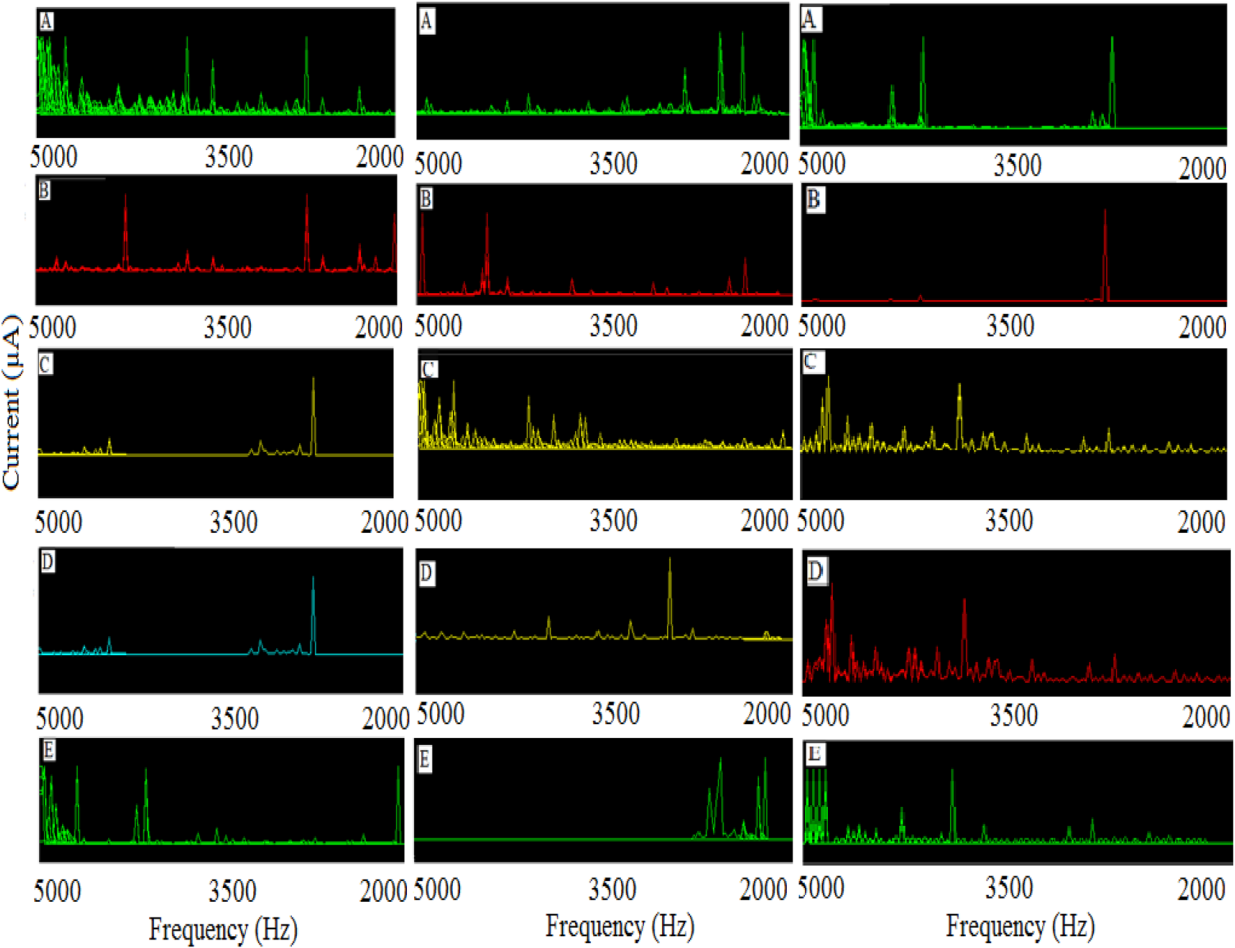
Spectra of (**A**) Real part, (**B**) Imaginary part, (**C**) Magnitude, (**D**) Phase and (**E**) Power spectrum for left column) *Cysticercus tenuicollis, middle column) Hydatid* cyst and right column) *C. cerebralis.* Triggered electrical potential: + 1.50 (± 0.01) V (vs. the pseudo reference probe). Time interval: 5.0 min. Inter-electrode distance: 0.50 cm, frequency step: 2 Hz.

Due to the dependency of the activity of the tested cysts to the electrical frequency, as well as because of the small sensitivity of the detection system, it was suggested to apply the *DFFT* algorithm to the trace (current–time) diagrams to access the related spectrum (i.e., the diagram of detector response versus the frequency at the selected triggered frequency using the three FIDs). According to the time domain as the selected model (Fig. 4), the spectra during analyses of *C. tenuicollis*, *Hydatid* cyst and *C. cerebralis*, under similar conditions, have been shown in Fig. 6.

As clearly shown (Fig. 6), significant changes were observed in different electric spectra depending on the type and structure of the tested cyst. These results can be utilized to approach to the introduction of reliable detection system for the cyst’s recognition process.

### Figures of merit

For better comparison, the figures of merit of the detection system have been summarized in Tables 3 and 4. These results were estimated based on the special frequencies detected based on the spectra shown in Figure 6.

**Table 3:** Selectivity of the bio-analysis system.

Cyst type	Cyst no	*Related frequency (kHz)			
		Real part	Imaginary part	Magnitude	Power spectrum
*C. tenuicollis*	1	3.5–5.0	2.0–3.5	2.0–3.5	3.5–5.0
*Hydatid cyst*		2.0–3.5	3.5–5.0	3.5–5.0	2.0–3.5
*C. cerebralis*		3.5–5.0	2.0–3.5	3.5–5.0	3.5–5.0

*Estimated based on direct interpretation of spectra shown in Fig. 6. The data ere interposed and reported based on at least two replicate analyses of the cyst by the recommended procedure under similar conditions. The data were reported based on both direct observations and evidenced by t-test at 95% confidence level.

**Table 4:** Some figures of merit of the designed detection system.

Cyst type	^1, 2^Maximum Sensitivity per each cyst (a.u.)		^3^Precision (RSD %, n = 3, reproducibility)	^4^Response time (min)	^5^Selectivity	^6^Effect of interference(s)
	Peak height	Peak area				
*C. tenuicollis*	3.27 ± 0.11	19.48 ± 0.13	3.05 ± 0.05	5.0	Specific differentiation system	No significant interference(s)
*Hydatid cyst*	4.13 ± − 0.08	27.02 ± 0.12	2.61 ± 0.07			
*C. cerebralis*	5.86 ± 0.12	34.15 ± 0.09	1.19 ± 0.05			

^1^Estimated based on the most intensive peak according to Fig. 6.

^2^Calculated based on maximum peak height and peak area of the selected frequency peaks.

^3^Percenatge of relative standard deviation.

^4^Estimated based time interval needed to access to at least three sequential FIDs.

^5^Estimated via comparison between the frequencies during analysis of different cysts.

^6^Estimated based on comparison between the peak position of the spectra for different tested cysts. ± Relative standard deviation (n = 3), frequency step: 2 Hz.

Based on the results (Table 3), some special differences were observed during interaction between the cyst and the electric waves, depending on different aspects especially their type, structure, surface size and different blind/in-blind characteristics of the tested cysts under similar condition. Other Figures of merit have also been reported in Table 4.

As shown (Table 4), again different figures of merit have been recognized and estimated for each tested cyst that reveled their different interactions with the external field.

### Proposed mechanism (cyst mode): correlation between excess surface charge, Zeta potentials and the peak area of the frequency domains

To better interpret the proposed mechanism (mode) of the interaction between the cysts and electrical wave, the correlation between different figures of merit and the excess surface charge (that was in good correlation with Zeta potential)^40^ have been evaluated in detail as shown in Table 5.

**Table 5:** Zeta potentials of different tested cysts before and after VLF irradiation.

Sample no	Cyst type	^1^Zeta potential (mV, vs. the pseudo reference probe)	
		Before voltage triggering (Fresh sample)	After VLF irradiation
1	*C. tenuicollis*	−2.03 ± 0.09	−6.05 ± 0.07
2	*Hydatid cyst*	−2.23 ± 0.08	−15.17 ± 0.09
3	*C. cerebralis*	−2.26 ± 0.09	−34.53 ± 0.12

^1^Analyzed using the Zeta meter instrumentation system according to the recommended procedure under similar conditions. ± Relative standard deviation (n = 3).

Based on the results (Table 5), no significant differences were observed in the excess surface charge, which was in good correlation with the *Zeta* potentials of the tested cysts under similar conditions. Whereas major gradients were recognized after irradiating (triggering) the tested cyst with the VLF waves according to the recommended procedure. This event pointed to the presence of significant interaction(s) between the different excess electrical charges of the cysts and selected VLF waves. As conclusion, this phenomenon was considered as the light view point for better cyst recognition and more efficiently treat with them.

## Conclusion

Comparative electric behavior of *C. tenuicollis*, *Hydatid cyst* and *C. cerebralis* at the VLF region has been studied in detail. The significance of this investigation is related to the importance of the detection of the above-mentioned cysts in the body of animals and human beings. In this report, single cell signal recording analysis has been adopted as the selected methodology for the cysts detection using a two- electrode system, implanted onto the cyst tissue at the giga ohm sealed condition.

The selectivity as well as the reliability of this bio-analysis system was evaluated via (i) accessing to a fixed frequency range, (ii) based on comparison between these three cysts (at least 15 samples of each cyst), (iii) repeatability of this detection system during sequential analyses of each selected cysts, (iv) possibility to access a reproducible condition during averaging the VLF scanning process and finally, (v) presence of good correlation between the results and the Zeta potentials. This bio-detection system is considered as a basis for approaching the *in- vivo* sensing process via correlation between the electrical waves, inductive magnetic field and the cysts. To the best of knowledge, no report has been introduced in the literature about this issue. It is hoped that this study could be a base for simpler and rapid *in vivo* diagnosis of different cysts in animals as well as human beings. In addition, we hope this study would open new horizons for *in vivo* treatment of the infection in the intermediate hosts in future via induction of electromagnetic field in the infected organs. However, obviously, long way forward is ahead to access the ideal conditions for the reliable detection as well as location of the cysts in the body of animals and human being.
